# Attenuating human fear memory retention with minocycline: a randomized placebo-controlled trial

**DOI:** 10.1038/s41398-024-02732-2

**Published:** 2024-01-17

**Authors:** Yanfang Xia, Jelena Wehrli, Aslan Abivardi, Madalina Hostiuc, Birgit Kleim, Dominik R. Bach

**Affiliations:** 1 Computational Psychiatry Research, Department of Psychiatry, Psychotherapy and Psychosomatics, Psychiatric University Hospital Zurich, University of Zurich, Zurich, Switzerland; 2 Transdisciplinary Research Area Life and Health, Hertz Chair for Artificial Intelligence and Neuroscience, University of Bonn, Bonn, Germany; 3 Wellcome Centre for Integrative Neuroimaging, FMRIB, Nuffield Department of Clinical Neurosciences, University of Oxford, Oxford, UK; 4 Wellcome Centre for Human Neuroimaging & Max Planck UCL Centre for Computational Psychiatry and Ageing Research, University College London, London, UK

**Keywords:** Human behaviour, Psychiatric disorders, Learning and memory

## Abstract

Pavlovian fear conditioning is widely used as a pre-clinical model to investigate methods for prevention and treatment of anxiety and stress-related disorders. In this model, fear memory consolidation is thought to require synaptic remodeling, which is induced by signaling cascades involving matrix metalloproteinase 9 (MMP-9). Here we investigated the effect of the tetracycline antibiotic minocycline, an inhibitor of MMP-9, on fear memory retention. We conducted a pre-registered, randomized, double-blind, placebo-controlled trial in *N* = 105 healthy humans (*N* = 70 female), using a configural fear conditioning paradigm. We administered a single dose of minocycline before configural fear memory acquisition and assessed fear memory retention seven days later in a recall test. To index memory retention, we pre-registered fear-potentiated startle (FPS) as our primary outcome, and pupil dilation as the secondary outcome. As control indices of memory acquisition, we analyzed skin conductance responses (SCR) and pupil dilation. We observed attenuated retention of configural fear memory in individuals treated with minocycline compared to placebo, as measured by our primary outcome. In contrast, minocycline did not affect fear memory acquisition or declarative contingency memory. Our findings provide in-vivo evidence for the inhibition of fear memory consolidation by minocycline. This could motivate further research into primary prevention, and given the short uptake time of minocycline, potentially also secondary prevention of PTSD after trauma.

## Introduction

After exposure to psychological trauma, 17–29% of sufferers develop post-traumatic stress disorder (PTSD) [1]. Worldwide, the prevalence of PTSD is 13–20% in women and 6–8% in men [2]. Currently recommended treatments involve trauma-focused cognitive behavior therapy [3], eye movement desensitization and reprocessing [4], and pharmacotherapy [5]. However, about one-third to half of the patients remain symptomatic after treatment [6–8]. The prognosis of PTSD could be improved by developing targeted interventions to prevent consolidation, reconsolidation, or return of traumatic memory after extinction [9–12].

Memory consolidation, in particular, relies on structural synaptic plasticity [13, 14], which is elicited by signaling cascades involving matrix metalloproteinase 9 (MMP-9) [15, 16]. As an inhibitor of MMP-9 [17–20], the tetracycline antibiotic minocycline might have potential to interfere with memory consolidation. Such properties have been reported for the chemically related doxycycline for cued fear conditioning [21] but with mixed results in more realistic pre-clinical models [22]. Minocycline, in comparison to doxycycline, has higher specificity for MMP-9 [17] and penetrates the blood-brain barrier more rapidly [23]. In addition to inhibiting MMP-9, minocycline also interferes with microglia [24, 25], which modulate synaptic consolidation [26]. This and its potential neuroprotective effects have sparked interest in its use for treating neurodegenerative disease. In aged animals and in Alzheimer’s disease models [27–29], prolonged minocycline treatment has been shown to improve hippocampus-dependent spatial memory. However, this improvement is not observed in non-aged healthy animals or humans [30]. In the latter study, in fact, prolonged minocycline treatment markedly reduced landmark-based spatial memory [30], in line with its potential effect on synaptic consolidation. Whether acute administration of minocycline impacts on spatial or aversive memory consolidation, however, remains unclear.

Pavlovian fear conditioning is a form of aversive learning and is regarded as a pre-clinical model of PTSD [31]. It is widely used to investigate potential interventions to improve PTSD treatment [32]. In this paradigm, initially neutral cues (conditioned stimuli, CSs) predict the occurrence or absence of an aversive stimulus (unconditioned stimulus, US), usually an electric shock. While the most basic implementation of Pavlovian fear conditioning uses single-feature CS, this might not be a realistic model of traumatic memory, which can be evoked by the context previously surrounding the traumatic event. Thus, in the present study, we used a presumably hippocampus-dependent configural fear conditioning paradigm [33–35] and investigated the effect of minocycline on configural memory retention. We identified the optimal quantification of memory retention in this paradigm in a preceding methodological investigation [36]. Accordingly, we pre-registered fear-potentiated startle eye-blink responses (FPS) during configural memory recall as primary outcome, and pupil dilation as secondary outcome. To control for an impact of minocycline on initial acquisition, we quantified learning by skin conductance responses (SCR) and pupil dilation during acquisition.

## Methods and materials

### Participants

#### Overview

**Fig. 1:**
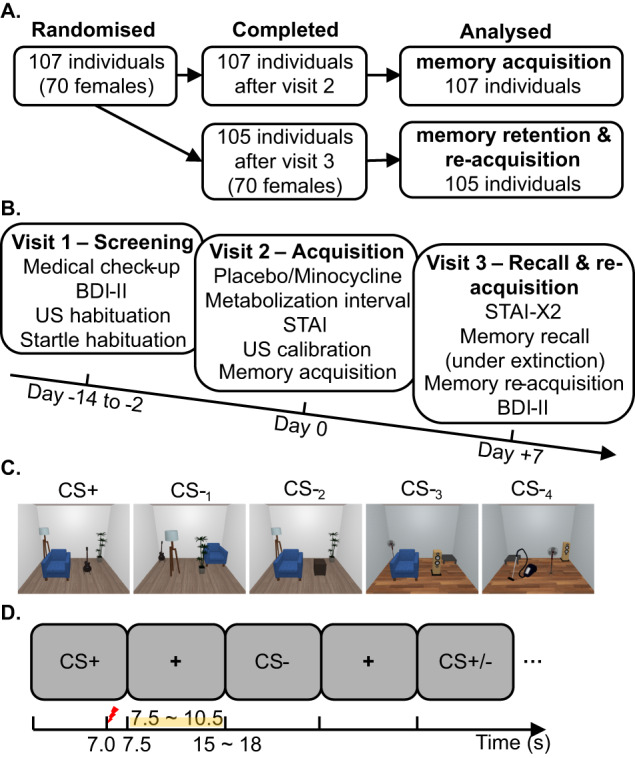
Experimental protocol. **A** Recruitment and inclusion of participants. **B** Study visit timeline. **C** Overview of CS images. **D** Intra-trial procedure in the acquisition training. A CS image was presented for 7.5 s; 83% of the CS+ (five out of six in each block; in total four blocks) co-terminated with a 0.5-s US (painful electric stimulation). No startle probe was delivered during acquisition. CS conditioned stimulus, US unconditioned stimulus, BDI-II Beck Depression Inventory-II, STAI State-Trait Anxiety Inventory.

We recruited 107 healthy volunteers from the general population by advertisements online and in public places between 13 July 2021 and 7 July 2022. Within either of the biological sexes, each individual was randomly assigned to placebo (*N* = 54, 35 females) or minocycline (*N* = 53, 35 females). Two male participants (one minocycline, one placebo) did not attend the recall test in visit 3. The reported final sample, therefore, includes 107 participants for the acquisition training and 105 for the recall test and the re-acquisition training (Fig. 1A). The two groups did not differ in age, sex, body mass index, baseline personality measures, and metabolization interval (Table 1). Individually calibrated US intensity was lower in the minocycline group than in the placebo group (see Supplementary Information “unconditioned stimulus” for US intensity determination) and was used as a covariate in robustness analyses. All participants were screened for in- and exclusion criteria by a study physician (see Supplementary Information).

**Table 1:** Group characteristics.

	minocycline	placebo	*p*	*d*
Sex	35 females, 18 males	35 females, 19 males	0.894	—
Age	23.28 ± 3.76 yrs	23.06 ± 2.91 yrs	0.727	0.07
BMI	21.86 ± 2.31	22.41 ± 3.10	0.302	−0.20
US intensity	4.08 ± 1.34 mA	5.02 ± 2.24 mA	**0.010**	−0.51
Pain difference	−8.94 ± 14.13	−7.08 ± 10.84	0.447	−0.15
Metabolization interval	127.25 ± 11.16 min	126.28 ± 9.88 min	0.636	0.09
STAI X1 pre-acquisition	32.60 ± 7.92	33.06 ± 7.44	0.761	−0.06
STAI X2 pre-acquisition	30.62 ± 5.96	31.78 ± 6.48	0.340	−0.19
STAI X2 pre-recall	29.48 ± 6.97	30.98 ± 6.25	0.248	−0.23
BDI pre-acquisition	3.11 ± 3.55	2.91 ± 2.78	0.739	0.06
BDI post-re-acquisition	3.52 ± 4.17	4.28 ± 3.95	0.338	−0.19
Drug guess	16 minocycline	16 minocycline	—	—

*BDI* Beck Depression Inventory-II, *BMI* body mass index, *US intensity* electric current of pain stimulus used as US in mA, *STAI* State-Trait Anxiety Inventory (X1: trait anxiety; X2: state anxiety); Pain difference, the difference in averaged pain ratings of the 14 US calibration stimuli before and after the acquisition training (see section “unconditioned stimulus”); Metabolization interval, the actual duration between drug ingestion and the start of the acquisition training; Drug guess, participants’ guess on whether they took minocycline or placebo; *p* *p*-values of Pearson’s Chi squared test (for sex distribution) or unpaired t-tests between the two groups (uncorrected for multiple comparison), bold type: *p* < 0.05; d, Cohen’s d of the difference between the two groups. One male participant in the minocycline group and one male in the placebo group did not attend the recall test and the re-acquisition training, and the rating data of the US stimuli before the acquisition training of one female participant in the placebo group were missing.

The study was conducted according to the Declaration of Helsinki and approved by the governmental research ethics committee (Kantonale Ethikkomission Zürich, KEK-ZH 2020-02944) and the Swiss Agency for Therapeutic Products (Swissmedic, Bern, Switzerland; 2021DR4072). All participants gave written informed consent with a form approved by the ethics committee. The study was pre-registered at a WHO-approved primary registry (German Clinical Trials Register, DRKS00024001) and in the Swiss Federal Complementary Database (Kofam: SNCTP000004350).

#### Power analysis

We conducted a power analysis in G*power [37] to determine the required sample size based on the effect size of d = 1.17 for CS + /CS- FPS difference in a control group in a methodological work [38]. Under the best-case assumption of this effect size, equal variance in the minocycline group, and no variation in the treatment effect [39], a 50% reduction in fear memory retention indexed by FPS would correspond to an effect size of d = 0.59. Thus, a sample size of *N* = 74 would be required to acquire 80% power at an alpha rate of 0.05 in a one-tailed t-test. To account for unknown treatment effect variability and allow for early dropouts, early withdrawal, and data exclusion, we planned to recruit *N* = 100 participants.

The effect sizes for this power analysis were based on a study using cued delay fear conditioning [38]. After the trial protocol was registered, we derived effect sizes for the CS + /CS- difference in a methodological study [36] using the same configural fear conditioning paradigm as in the current trial. This study revealed a smaller effect size of d = 0.71 for the CS + /CS- FPS difference. Using this effect size estimate, a 50% reduction in fear memory retention indexed by FPS would correspond to an effect size of d = 0.36. In consequence, we continued recruitment beyond the target sample size as long as logistic constraints allowed. Post hoc, with a sample size of *N* = 107, our power to detect a 50% reduction in fear memory at an alpha rate of 0.05 in a one-tailed t-test was 58%. Notably, our pre-registered primary analysis was not based on t-tests but on linear mixed effects (LME) models, but there is no established framework for power analysis with this statistical model.

### Study medication

The study medication was minocycline, Minocin (Drossapharm AG, Basel, Switzerland), and the placebo was mannitol. We used a dose of 200 mg based on the smallest antibiotic starting dose for adults, according to the manufacturer’s recommendation [40], to minimize side effects. The peak serum concentration of minocycline is reached approximately 120 min after oral administration [41], and the penetration ratio of cerebrospinal fluid to serum concentration is between 15% and 65% [42]. The half-life of minocycline is 12–17 h [41], such that the drug was cleared by more than 99.9% during the recall test 7 days after acquisition. A GMP-licensed pharmacy (Kantonsapotheke Zürich, Switzerland) manufactured, blinded and randomized the study medication separately for males and females. Randomization was broken after the last visit of the last participant and after ensuring data consistency. Three participants in each group reported adverse events (placebo: cold, tiredness, and nausea; minocycline: nausea). No participant withdrew from the study due to adverse events, and there were no serious adverse events.

### Study procedure

#### Visit 1 – Screening (day -14 to day -2)

The study procedure is depicted in Fig. 1B. The study physician screened participants medically, checked in- and exclusion criteria (see Supplementary Information), and measured weight/height. Further, we screened participants’ mental health using Beck’s Depression Inventory [43] and habituated them to electric shocks (US) and startle probes.

#### Visit 2 – Acquisition training (day 0)

Visit 2 took place in the morning between 0815 and 1330 h. Participants were asked about their health status, medication and substance intake since visit 1 before ingesting the study medication. During the first 60 min of the 120-min metabolization interval, they were not allowed to eat or drink and were kept under the surveillance of study staff. During the last 30 min of the interval, participants filled in the German or English version of the state-trait anxiety inventory (STAI) [44, 45], and we calibrated US intensity (see details in Supplementary Information). Fear acquisition training started approximately 120 min after drug ingestion (Table 1 for actually realized intervals).

The training protocol was a configural conditioning paradigm adapted from Stout et al. [33, 34] and used in our previous methodological work [36]. The paradigm consisted of 88 trials in four blocks (six CS+ and 16 CS- in each block; Fig. 1D), with self-paced breaks between blocks. CSs were five static room images containing four furniture items (see Fig. 1C and Supplementary Information Figure S1 for the detailed configuration of CSs), and one of the images (CS + ) co-terminated with an electric stimulation as the aversive US in five out of six CS+ trials in each block. The remaining four images served as CS-; four different CS- were implemented to reduce fear conditioning towards elemental cues. No startle probes were delivered during the acquisition training to avoid their potential influence on differential fear learning as has been demonstrated using pupil dilation [46], SCR and verbal ratings [47]. Within each block, trials were pseudo-randomized: the first ten trials consisted of two trials of each CS in random order, with the first CS+ trial always reinforced. The remaining 12 trials consisted of four CS+ and two trials of each of four CS- in random order. During acquisition training, CS images were presented full screen for 7.5 s, and a gray (RGB: [178.5, 178.5, 178.5]) background was presented during the inter-trial interval (ITI). ITI was randomly drawn from {7.5 s, 8.5 s, 9.5 s, 10.5 s}. CS + /CS- assignment was the same for every participant. To maintain participants’ attention during training, they were instructed to press the “DOWN” arrow key as soon as they thought they had identified the specific room layout in each trial.

Immediately after acquisition training, participants gave ratings for arousal, valence, and CS-US contingency to each CS. Subsequently, participants received the US calibration stimuli with the same intensities as in the pre-acquisition determination in random order and rated each intensity.

#### Visit 3 – Recall test and re-acquisition training (day + 7)

Visit 3 took place seven days after the acquisition training. Participants were asked for adverse events following visit 2, gave their guesses on which drug they took in visit 2, and filled out the state part of the STAI. They received the same instructions about the US as in visit 2, and US electrode was attached to the same position as in visit 2. Before the recall test, participants were exposed to eight startle probes with an inter-stimulus interval of 4 s. The recall protocol was the same paradigm as in visit 1 with 88 trials in four blocks; no US was delivered during the recall test, while a startle probe was delivered at the expected time point of the US in all trials (i.e., CS+ and CS-). After the recall test, participants again rated arousal, valence and CS-US contingency for each CS in the recall test, as well as the remembered CS-US contingency in visit 2. A re-acquisition training followed immediately after the ratings and consisted of 44 trials in two blocks. The settings were the same as the acquisition training, with US reinforcement but without startle probes. After the re-acquisition training, the participants again rated arousal, valence and CS-US contingency, followed by ratings on US calibration stimuli in random order and the BDI questionnaire.

### Psychophysiological modeling

We pre-registered our analysis plan on OSF (10.17605/OSF.IO/CZHP7) on 15 July 2022 before breaking the randomization of the group assignment. All data pre-processing and model-based analysis were conducted using the PsPM toolbox (version 5.1.1, bachlab.github.io/pspm) in Matlab (R2018b, The Math Works, Natick, MA, USA). We recorded and analyzed skin conductance responses (SCR), fear-potentiated startle eye-blink responses (FPS) and pupil diameter (see recording details, data pre-processing and psychophysiological modeling in Supplementary Information).

### Statistical analysis

#### Outlier rejection and general information

Twelve participants were excluded from pupil dilation analysis for memory retention due to raw data quality (see Supplementary Information). Excluding the same participants from FPS analysis did not change our results. To control data quality after pre-processing, we extracted estimates of individual trials in each CS condition in temporal sequence, and averaged data across participants in each group. For each trial, data outside three standard deviations around the group mean of this condition were excluded. No participant was completely excluded by this procedure. The final sample size included in each analysis is summarized in Table S1 in the Supplementary Information. In brief, we retained more than 98.8% of trials for FPS and SCR analysis and more than 89.9% of trials for pupil size.

We conducted statistical analyses in R (version 4.1.0, www.r-project.org) for linear mixed effects models (LME, function lmerTest::lmer()) and repeated-measures ANOVA (rmANOVA, function aov()). Greenhouse-Geisser correction for lack of sphericity (rstatix::anova_test()) was applied whenever needed. Post-hoc two-tailed t-tests were computed in Matlab (R2018b). Effect sizes for rmANOVA were calculated as partial eta squared (µ^2^; rstatix::anova_summary()) [48] and for t-tests as Cohen’s d [49].

According to the pre-registered analysis plan (10.17605/OSF.IO/CZHP7) based on our prior methodological work [36] with the same experimental settings, we quantified configural fear retention by differential FPS (primary outcome) and pupil dilation (secondary outcome). To corroborate that learning took place in the acquisition session and investigate potential drug effects on acquisition and re-acquisition, we quantified fear acquisition and re-acquisition by pupil dilation and CS onset-evoked SCR. To avoid US contamination, we only included non-reinforced trials in the latter analyses, while reinforced CS+ trials were treated as missing data. To confirm robustness of our results to sex, we conducted stratified analyses, separately for each sex (see Supplementary Information Table S9-12 for results).

#### Recall test

As primary analysis, we conducted an LME analysis of FPS estimates (primary outcome) and pupil dilation (secondary outcome), across trials (numeric; linear trial index), drug (2 levels; placebo/minocycline) and conditions (5 levels; CS + /CS-_1-4_) with a random effect for participants (formula: data ~ drug*conditions*trial index + (1/subject)), to account for the main and interaction effects of time (e.g., the dynamic learning process and response habituation). The trial index was not mean-centered, i.e., when there is an interaction of any variable with trial index then all lower-order effects should be interpreted as relating to the beginning of the session. As a robustness analysis based on US intensity differences between the groups, we added US intensity and its interaction with condition as a covariate into the LME models. This did not change any results and revealed no main effect of US intensity or interaction with conditions. All results are therefore reported as pre-registered, without the covariate. As secondary analysis, we conducted t-tests on CS + /CS- differences as described in the following. Notably, only the first ten trials were fully randomized and balanced in CS + /CS- conditions, consisting of two trials of each CS. To avoid impacts of response habituation on the condition averages, we sorted trials in each block into four subsets. The first two subsets comprise the first or the second trial of each CS, respectively. Because the recall test was conducted without reinforcement, those participants who did retain differential fear memory are a priori expected to extinguish this differential memory during the recall test. Hence, it is expected that the difference between the drug and placebo group would be most pronounced early in the recall test. As pre-registered, we computed paired t-tests on CS + /CS- differences for the first and the average of the first two subsets in each group and two-sample unpaired t-tests for between-group differences for the first and the average of the first two subsets. Group comparisons from this analysis were Holm-Bonferroni corrected for two comparisons (i.e., the first subset and the average of the first two subsets).

#### Acquisition and re-acquisition training

We averaged over trials for each condition for pupil dilation and fixed-latency SCR estimates. Estimates of the four CS- conditions were averaged to yield an overall estimate on CS-. As primary analyses, we conducted paired t-tests on CS + /CS- differences within each experimental group and two-sample unpaired t-tests for between-group comparison of CS + /CS- differences. As secondary analyses, we conducted LME analyses using the same formula as in the recall test. Robustness analyses with US intensity and its interaction with condition as covariate in the LME models did not change any results and revealed no interaction with conditions, and with one exception, no effect of US intensity. Only for SCR in re-acquisition training we observed a main effect of US intensity. All results are therefore reported as pre-registered, without the covariate.

#### Subjective ratings

We analyzed subjective ratings (i.e. arousal, valence, and CS-US contingency) in a 2 (drug) × 5 (CSs) × 3 (rating time: post-acquisition/post-recall / post-re-acquisition) multi-stratum rmANOVA (formula: data ~ drug*rating time*conditions + Error(subject/(drug*rating time*condition))). As an explorative post-hoc analysis based on the rmANOVA result, we conducted an unpaired two-sample two-tailed t-test on differential CS ratings of valence after acquisition.

## Results

### Fear retention

**Fig. 2:**
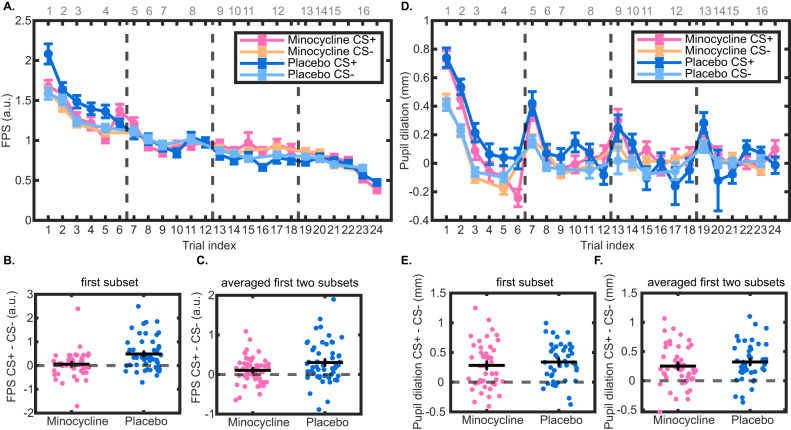
Configural fear memory retention in the recall test seven days after acquisition training. **A** Normalized trial-wise FPS and the averaged FPS over **B** the first and **C** the first two subsets. **D** Trial-wise pupil dilation and the averaged pupil dilation over **E** the first and **F** the first two subsets. “CS-” represents the averaged data over all four CS- conditions. Data shown in **A** and **D** are the group means ± SEM of each trial in each condition. Data points in the figure were generated by averaging FPS estimates across the time sequences of the semi-randomized trials in each condition of all participants. The top of the plots shows the trial indices of CS- conditions, and the bottom shows the indices of CS + . The black dashed lines separate trials into four blocks, with six trials of CS+ and four trials of averaged CS- in each block. Data shown in **B**, **C** and **E**, **F** are the group means ± SEM of the difference between CS+ and averaged CS- over all four CS- conditions.

LME analysis of FPS, our primary analysis and outcome, showed attenuated fear retention in the minocycline group (drug × condition interaction, Table 2) and more pronounced extinction in the placebo group (drug × trial × condition). Furthermore, the minocycline group showed overall lower responses (main effect drug) and less pronounced habituation (drug × trial index). Across both groups, we observed fear retention (main effect condition) and habituation (main effect trial, see Supplementary Information Table S2). As secondary analysis, unpaired t-tests showed attenuation of conditioned memory retention in the minocycline group, compared to placebo, by more than 85% for the first subset of trials (t (101) = −3.62, *p* < 0.001, d = −0.71) and more than 60% for the average of the first two subsets (t (103) = −2.33, *p* = 0.022, d = −0.46; Fig. 2B, C). Participants in the placebo group retained fear memory (first subset: t (51) = 5.26, *p* < 0.001, d = 0.73, first two subsets: t (52) = 4.32, *p* < 0.001, d = 0.59) (see Supplementary Information Table S3 for the paired t-test results of the minocycline group).

**Table 2:** LME results for recall test and fear acquisition.

	**Fear recall test**					
	**FPS**			**Pupil dilation**		
Effect	F	df	*p*	F	df	*p*
Drug (placebo/minocycline)	8.47	1, 9115	**0.004**	0.11	1, 237.5	0.744
Trial index	2689.17	1, 9115	**<0.001**	105.54	1, 7345.1	**<0.001**
Condition (CS + /CS-_1,2,3,4_)	8.94	4, 9115	**<0.001**	11.03	4, 7318.3	**<0.001**
Drug x Trial index	12.52	1, 9115	**<0.001**	0.98	1, 7345.1	0.322
Drug x Condition	3.39	4, 9115	**0.009**	1.53	4, 7318.3	0.192
Trial index x Condition	7.41	4, 9115	**<0.001**	3.31	4, 7319.4	**0.010**
Drug x Trial index x Condition	3.12	4, 9115	**0.014**	1.14	4, 7319.4	0.335
	**Fear acquisition training**					
	**Pupil dilation**			**Fixed-latency SCR**		
Effect	F	df	*p*	F	df	*p*
Drug (placebo/minocycline)	4.93	1, 425.3	**0.027**	33.54	1, 7021	**<0.001**
Trial index	89.38	1, 5942.8	**<0.001**	832.74	1, 7021	**<0.001**
Condition (CS + /CS-_1,2,3,4_)	16.57	4, 5922.9	**<0.001**	9.71	4, 7021	**<0.001**
Drug × Trial index	6.25	1, 5942.8	**0.012**	48.66	1, 7021	**<0.001**
Drug × Condition	1.81	4, 5922.9	0.124	0.13	4, 7021	0.970
Trial index × Condition	0.60	4, 5924.7	0.661	2.39	4, 7021	**0.049**
Drug × Trial index × Condition	1.19	4, 5924.7	0.312	0.71	4, 7021	0.583

The effect "Drug x Condition" relates to our primary hypothesis.

LME analyses of pupil dilation, our secondary outcome, showed no effects involving drugs (Table 2, Fig. 2D). Unpaired t-tests also suggested no group difference (first subset: t (84) = −0.73, *p* = 0.653, d = −0.16; first two subsets: t (87) = −0.99, *p* = 0.653, d = −0.21). Participants in the placebo group retained fear memory (first subset: t (41) = 6.85, *p* < 0.001, d = 1.06; first two subsets: t (43) = 6.61, *p* < 0.001, d = 1.00) (Fig. 2E, F) (see Supplementary Information Table S3 for the paired t-test results of the minocycline group).

### Configural fear acquisition

**Fig. 3:**
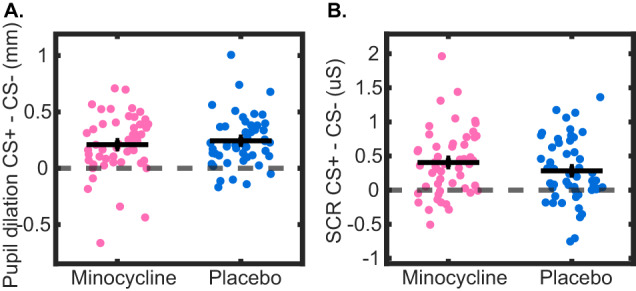
Configural fear memory acquisition. **A** Pupil dilation and **B** normalized CS onset-evoked SCR in the acquisition training. Only data from non-reinforced trials are included. Black crosses represent the group means ± SEM of the difference between CS+ and averaged CS- over all four CS- conditions.

Participants acquired differential memory under both placebo and minocycline as quantified by pupil dilation (paired t-test, placebo: t (47) = 7.33, p < .001, d = 1.06; minocycline group: t (50) = 5.68, *p* < 0.001, d = 0.79) and CS onset-evoked SCR (placebo: t (51) = 4.38, *p* < 0.001, d = 0.61; minocycline: t (52) = 6.22, *p* < 0.001, d = 0.85; Fig. 3). There was no evidence for a group difference in memory acquisition in unpaired t-tests, as indexed by pupil dilation (t (97) = −0.69, *p* = 0.49, d = −0.14) and SCR (t (103) = 1.34, *p* = 0.18, d = 0.26). Secondary LME analysis of pupil dilation revealed learning of the CS + /CS- difference (main effect condition), response habituation (main effect trial), overall lower responses in the minocycline group (main effect drug) as well as reduced habituation in the minocycline group (interaction drug x trial index, Table 2, Supplementary Information Figure S2A-C, Table S4). Similarly, LME analysis of SCR revealed learning of the CS + /CS- difference (main effect condition and trial x condition interaction), response habituation (main effect trial), overall lower responses in the minocycline group (main effect drug), as well as reduced habituation in the minocycline group (interaction drug x trial; Table 2, Supplementary Information Fig. S2D-F, Table S5).

### Conditioned configural memory re-acquisition

Re-acquisition training took place immediately after the recall test. Both groups successfully re-acquired conditioned configural memory without main effect or interaction involving drug (see Supplementary Information Table S6-7 for statistical results and Figure S3 for illustration).

### Subjective ratings

Minocycline did not affect subjective ratings on arousal, valence and CS-US contingency (see Supplementary Information Figure S4 and Table S8). We observed a reduction of positive evaluation towards CS- after the acquisition training in the minocycline group (interaction of drug × rating time in the rmANOVA of valence rating, *p* = 0.044; explorative post-hoc t-test: t(103) = 2.10, *p* = 0.039, d = 0.41).

### Stratified analyses in females and males

We conducted stratified robustness analyses for female and male participants separately (see Supplementary Information Table S9-12). Notably, FPS showed minocycline-induced attenuation in memory retention in female participants, but not in male participants.

## Discussion

Improving PTSD prevention and treatment strategies is a priority. Capitalizing on Pavlovian fear conditioning as a pre-clinical model in healthy humans, we investigated whether the MMP9 inhibitor and microglia modulator minocycline affects configural fear memory consolidation. We observed attenuated memory retention in participants who acquired fear memory under minocycline, compared to those under placebo, as quantified by fear-potentiated startle. There was no evidence that minocycline affected overall memory acquisition, suggesting that results were due to an impact on consolidation rather than encoding, with the caveat that our study was not designed to conclusively address this point. Robustness analyses confirmed the results of our primary analyses. Notably, there was no effect of the drug on the secondary outcome measure, pupil dilation, in the recall test.

Previous work addressed the impact of the chemically similar tetracycline antibiotic doxycycline on different forms of Pavlovian fear memory. Doxycycline appeared to affect consolidation (and/or potentially encoding) of cued delay fear memory [21]; however, evidence for an effect on cued trace fear memory was ambiguous [22], and we found weak evidence against an impact on configural fear memory in the same paradigm used here [50]. Our motivation for testing minocycline in the present study was that compared to doxycycline, it inhibits MMP9 activity more effectively [17], and we reasoned that it might therefore have a clearer impact. Furthermore, doxycycline pharmacokinetics are not ideal for potential use in secondary prevention after trauma, as it may take 3-4 hours to reach peak cerebrospinal fluid (CSF) concentration [51, 52]. In contrast, minocycline penetrates the blood-brain barrier in larger quantities and more quickly [23]. Additionally, minocycline inhibits microglia activity [24, 25]. Both MMP9 and microglia contribute to synaptic consolidation of memory [26, 53, 54]. By interacting with multiple mechanisms [55], minocycline might provide a higher potential to influence memory consolidation. As a limitation, in contrast to a previous report [22], we did not measure the concentration of minocycline levels in the current study, nor did we record neural activity under minocycline treatment during configural fear conditioning and the recall test. Thus, further studies are needed to unravel the underlying mechanism of minocycline’s effect on fear conditioning.

Notably, we observed a reduction of fear memory retention in our primary (FPS) but not in the secondary (pupil dilation) outcome. There are several plausible explanations for this discrepancy. First, FPS is a well-established measure of fear memory retention across different mammal species and in various paradigms [56]. For example, interventions to reduce fear memory reconsolidation that were validated with FPS in healthy humans, such as propranolol [57], have tended to generalize to clinical populations [58]. On the other hand, fear-conditioned pupil dilation is much less well understood and has mainly been reported during acquisition rather than in a recall test [56]. Also, startle probes during the recall test might have an unintended impact on pupil responses. This is why FPS was pre-registered as the primary outcome, even though pupil dilation showed a higher effect size in a preceding methodological study [36]. Second, it has previously been argued that while several conditioned responses differentiate CS+ and CS- when averaged across all trials, they may index different components of the learning process and, therefore, could be differentially amenable to pharmacological intervention [56]. For example, it has been suggested that pupil dilation reflects US prediction during early learning but the uncertainty of US prediction at later stages [56, 59], or even general emotional arousal [60], which would endow pupil size estimates with a different interpretation during a recall test compared to acquisition training. Another third possible reason is that the measurement of pupil dilation is less robust, especially in the current paradigm that necessitated eye movements.

While we did not observe any minocycline effect on memory acquisition when quantifying learning across all acquisition trials, LME analysis to account for the dynamic learning process suggested different patterns in the minocycline-treated participants compared to those under placebo. In particular, we observed delayed habituation in pupil dilation and SCR; however, these were not specific to CS+ or CS- and thus did not reflect on differential fear acquisition. Based on these analyses, it appears more likely that minocycline mainly impacts on consolidation, but we cannot conclusively rule out an impact on acquisition either.

The minocycline group received somewhat lower US intensity than the placebo group; however, adding US intensities as a covariate in the LME models in a robustness analysis did not change our results. Additionally, the fact that no differential acquisition was observed speaks against this as an explanation for our main results.

Our stratified analyses showed a convincing effect of minocycline in female, but not male participants. Notably, the latter analyses only included *N* = 35 individuals, and this provides insufficient power to rule out that minocycline is effective in males, or to conclusively test for sex differences.

In summary, our study demonstrates that minocycline induced attenuation in configural fear memory retention after seven days when minocycline was ingested before fear acquisition. In the future, our results might have clinical implications. First, minocycline could potentially be used for primary prevention in individuals at risk of trauma exposure, such as firefighters, when administered prior to potential traumatic events. To enable clinical RCTs, pre-clinical studies should elucidate the most advantageous dosage regime, the time window during which minocycline is effective in reducing aversive learning, potential side effects on other forms of memory, and the effect of repeated administration. Second, if future pre-clinical studies confirm that minocycline is also effective in blocking aversive memory when taken after encoding (rather than before as in the current study), then minocycline could potentially even be used for secondary prevention immediately after trauma exposure. Finally, it remains to be explored whether minocycline can also inhibit fear memory reconsolidation, which would open up a potential treatment strategy. To our knowledge, minocycline has not been tested in other fear conditioning paradigms in humans. Future studies might address the inhibition or improvement of various fear conditioning paradigms with minocycline.

### Supplementary information

supplementary information

## Data Availability

All data are available in anonymized form on Zenodo (10.5281/zenodo.7601792). Matlab code to run the experiment, pre-registration of analysis, and analysis scripts are available from OSF (10.17605/OSF.IO/P8JTB).
