# Correction: Attenuating human fear memory retention with minocycline: a randomized placebo-controlled trial

**DOI:** 10.1038/s41398-024-02813-2

**Published:** 2024-02-14

**Authors:** Yanfang Xia, Jelena Wehrli, Aslan Abivardi, Madalina Hostiuc, Birgit Kleim, Dominik R. Bach

**Affiliations:** 1 Computational Psychiatry Research, Department of Psychiatry, Psychotherapy and Psychosomatics, Psychiatric University Hospital Zurich, University of Zurich, Zurich, Switzerland; 2 Transdisciplinary Research Area Life and Health, Hertz Chair for Artificial Intelligence and Neuroscience, University of Bonn, Bonn, Germany; 3 Nuffield Department of Clinical Neurosciences, Wellcome Centre for Integrative Neuroimaging, FMRIB, University of Oxford, Oxford, UK; 4 Wellcome Centre for Human Neuroimaging & Max Planck UCL Centre for Computational Psychiatry and Ageing Research, University College London, London, UK

**Keywords:** Human behaviour, Psychiatric disorders, Learning and memory

Correction to: *Translational Psychiatry* 10.1038/s41398-024-02732-2, published online 17 January 2024

In the Abstract, the full term for “FPS” should be “fear-potentiated startle”, but not “fear-potentially startle”.

The original article has been corrected.

