# Supplementary material for: Attenuating human fear memory retention with minocycline: a randomized placebo-controlled trial
Source: Transl Psychiatry. 2024 Jan 17;14:28. doi: 10.1038/s41398-024-02732-2 (PMC10794420; doi:10.1038/s41398-024-02732-2)
Supplement: Supplementary file 1 — supplementary information [file 41398_2024_2732_MOESM1_ESM.docx]

Supplementary Information for:

**Attenuating human fear memory retention with minocycline: a randomized placebo-controlled trial**

Yanfang Xia *, Jelena Wehrli *, Aslan Abivardi, Madalina Hostiuc, Birgit Kleim, Dominik R Bach

# Supplementary Information

Participant recruitment

CONSORT 2010 Flow Diagram

MinAv Trial

Enrolment

Assessed for eligibility

N = 128

Excluded (N = 21)

- Not meeting inclusion criteria (N = 13)
- Declined to participate (N = 5)
- Other reasons (N = 3)

Randomised

N = 107

Allocation

Allocated to intervention: Placebo (N = 54)

- Received allocated intervention (N = 54)
- Did not receive allocated intervention (N = 0)

Allocated to intervention: Minocycline (N = 53)

- Received allocated intervention (N = 53)
- Did not receive allocated intervention (N = 0)

Follow-up

Follow-up completed N = 105

Discontinued intervention N = 2 (no participation)

Analysis

Analysed: N = 107

Participant selection

Inclusion criteria:

1. Healthy
2. Aged between 18 and 40 years
3. Fluent speaking in German or English

Exclusion criteria:

1. Allergy to minocycline or any other ingredient in the named drugs
2. Use of any drugs in the two weeks before the study except for contraceptive drugs and incidental use of NSARs/NSAIDs or paracetamol
3. Women who are pregnant or breastfeeding
4. Intention to become pregnant during the course of the study
5. Lack of safe contraception, defined as: female participants of childbearing potential, not using and not willing to continue using a medically reliable method of contraception for the entire study duration and for up to two weeks after drug intake, such as oral, injectable, or implantable contraceptives, or intrauterine contraceptive devices, or a mechanical contraceptive (condom, diaphragm)
6. Other clinically significant concomitant disease states, e.g., renal failure (i.e., estimated glomerular filtration rate (eGFR; CKD-EPI) lower than 60 ml/min/1.73 m2), hepatic dysfunction (i.e., alanine transaminase (ALAT) higher than 90 U/I for women or 110 U/I for men, aspartate aminotransferase (ASAT) higher than 74 U/I, and/or gamma-glutamyl transferase (γGT) higher than 70 U/I for women or 120 U/I for men), cardiovascular disease, etc.
7. Any history of psychiatric, neurological, dependence or systemic/rheumatic disease
8. Known or suspected non-compliance, drug or alcohol abuse
9. Smoking more than five cigarettes per day
10. Inability to follow the procedures of the study, e.g., due to language problems
11. Participation in another study with an investigational drug within the 30 days preceding and during the present study
12. Previous enrolment into the current study
13. Previous enrolment into the methodological studies with the same experimental paradigm
14. Members of the study team and their family members and dependents

Blood parameters screened:

Blood cell count, electrolytes, C-reactive protein, estimated glomerular filtration rate (eGFR; CKD-EPI), aspartate aminotransferase (ASAT/GOT), alanine aminotransferase (ALAT/GPT), gamma-glutamyl transferase (γGT), creatinine, thyroid-stimulating hormone (TSH), free thyroxine (FT4)

Urine parameters screened:

Amphetamines, barbiturates, benzodiazepines, tetrahydrocannabinol, cocaine, methadone, opioids; women: beta human chorionic gonadotropin (beta-HCG) pregnancy test

Experimental instructions

Visit 2 - acquisition training (day 0)

“In this task, you will be presented with 5 different room images. Your task is to learn to differentiate the rooms as they share some similarities. You will be presented with all the room images before the experiment starts, so you can familiarize yourself with the stimuli. One of the rooms may occasionally be coupled with a shock, you should learn which of the rooms is associated with the shock throughout the experiment. For all pictures, no matter if it is linked to a shock or not, we ask you to press the key arrow for “DOWN” whenever a room picture is presented as soon as you identify the room layout of the room. During the experiment, we ask you to sit as still as possible and keep your eyes on the screen. We will record your psychophysiological responses during the experiment. There is no way for you to influence when a shock will be presented in the experiment, as this is determined randomly by the computer. Neither how quickly you react to the stimulus, nor your bodily response will influence the shock presentation. Shock presentation is only related to a specific room image. The experiment consists of four blocks; between the blocks, you may have a self-paced short break, please remain seated and don’t move at the pause. You may close your eyes to rest a bit during the pause. The whole experiment will take roughly 25 minutes. There is a camera and a microphone in the cabin, if necessary, you can call for me.”

Visit 3 – Recall test and re-acquisition training (day +7)

“In this task, you will be presented with 5 different room images you saw already in the last week. Your task is again to try and differentiate the rooms as they share some similarities. During the experiment, you may occasionally be presented with an electrical shock, but you will additionally also be presented with the white noise burst. The white noise bursts are delivered for study measurements only, will not influence shock presentation and have nothing to do with your learning. You don’t need to learn anything from the noises. Whenever a room picture is presented (no matter if it is a shock room or not), we ask you to press the key arrow for “DOWN” as soon as you identify the room layout. During the experiment, we ask you to sit as still as possible and keep your eyes on the screen. We will record your psychophysiological responses during the experiment. There is no way for you to influence when a shock will be presented in the experiment, as this is determined randomly by the computer. Neither how quickly you react to the stimulus, nor your bodily response will influence the shock presentation. The shock presentation is only related to the specific room image. The experiment consists of 6 blocks; between the blocks, you may have a self-paced short break, please remain seated and don’t move at the pause. You may want to close your eyes to rest a bit during the pause. Between the blocks, you may be presented with questionnaires similar to the ones last week. This does not mean that the experiment is finished. Please remain seated and on the headrest until I turn on the light and come into the cabin. The whole experiment will take roughly 35 minutes. There is a camera and a microphone in the cabin, if necessary, you can call for me.”

Stimuli and apparatus

Experiment presentation

The experiment was coded and displayed using the Cogent 2000 toolbox (v1.32, [www.vislab.ucl.ac.uk](http://www.vislab.ucl.ac.uk)) in Matlab (2019a, The Math Works, Natick, MA, USA) with a computer monitor (Dell P2014h, 20”, aspect ratio 4:3, resolution 1280 x 1024 pixels, refresh rate 60 Hz) in a dark and soundproof chamber. The participants positioned their heads on a chin rest in front of the monitor with an eye-monitor distance of 700 mm. Questionnaires were coded in OpenSesame (1).

Unconditioned stimulus

US was a 0.5-s electric shock of 250 square pulses with a 10% duty cycle. The US was delivered to the participants’ dominant forearm by a constant current stimulator (Digitimer DS7A, Digitimer, Welwyn Garden City, UK) with a pin-cathode/ring-anode configuration. The intensity of US was determined individually in a two-phase procedure: 1) a staircase procedure to approximate the pain threshold by gradually increasing intensities from unperceivable to painful in steps of 0.3-0.4 mA; 2) a random procedure to find the intensity used for the experiment by collecting self-ratings to 14 random electric stimuli (0% for no sensation and 100% for clearly painful). These random intensities corresponded to 40%-100% of the threshold established in the staircase procedure in 10%-steps; each intensity was rated twice in random order. A linear interpolation of the ratings and the intensities yielded the final intensity corresponding to 85-90% of the pain threshold. The intensity used for the acquisition and the re-acquisition training was the same and consistent throughout the tasks. Using the same intensities, the random procedure was repeated after acquisition and re-acquisition.

Startle probes

Startle probes were 40-ms white noise sounds of 102 dB loudness with an instantaneous rise time, delivered binaurally through headphones (HD 202, Sennheiser, Wedemark-Wennebostel, Germany).

Psychophysiological recordings

We recorded skin conductance responses (SCR) with two disposable pre-gelled Ag/AgCl snap electrodes (EL507, Biopac Systems Inc., Goleta, CA) and an additional layer of 0.5% NaCl gel (GEL 101, Biopac Inc.) (2) from the thenar/hypothenar of the non-dominant hand using a constant voltage coupler/amplifier (EDA100C, Biopac Systems Inc.). A ground electrode was attached to the non-dominant elbow. We recorded electromyogram (EMG) data from the left orbicularis oculi muscle through two 4-mm shielded Ag/AgCl cup electrodes (EL 254S, Biopac Systems Inc.) filled with highly conductive gel (SignaCreme 17-05, Parker Laboratories, Inc. USA). One electrode was placed centrally below the lower eyelid and the other one laterally below the canthus of the left eye with a center-to-center distance of 1-2 cm from the first one (3). EMG data were filtered with a band-pass filter of 1 and 500 Hz. SCR and EMG data were digitized at 2000 Hz (MP 160, Biopac Systems Inc.) and recorded with Acknowledge (v5.0, Biopac Systems Inc.).

Pupil diameter and gaze direction were recorded for both eyes with Eyelink 1000 System (SR Research, Ottawa, ON, CA) with a sampling rate of 500 Hz. The calibration and validation of gaze coordinates were conducted following the manufacturer’s nine-point protocol. The horizontal distance between the eye and the eye-tracking camera was 470 mm.

## Psychophysiological modelling

Fear-potentiated startle eye-blink responses (FPS)

We pre-processed EMG time series following the procedure in Khemka et al. (4) with optimized filter bands and pre-processing to maximize CS+/CS- difference in fear-potentiated FPS. Specifically, we used a fourth-order Butterworth low-pass filter with a cut-off frequency of 50-470 Hz, and a 50-Hz notch filter to remove mains noise. We then rectified and smoothed the data using a fourth-order Butterworth low-pass filter with a 3-ms time constant. We then visually inspected the pre-processed data for discernible FPS to startle probes averaged across all trials for quality control. No participant was excluded due to this control procedure. Next, we used a flexible latency GLM with a canonical response function embedded in PsPM (4) for FPS amplitude estimation. This yielded FPS amplitudes for each trial by linear regression onto a canonical response function, with variable onset of 0-150 ms after the recorded startle probe onsets. Lastly, we normalized individual FPS amplitude estimates for each participant by dividing them through the average of all CS- trials of the participant.

Skin conductance responses (SCR)

For SCR data, we visually inspected periods of missing data (data loss due to technical connection, clipping, etc.), artefacts, and non-discernible responses to the US. Two participants (female, placebo) were excluded from acquisition analysis and three (two females and one male, all placebo) from re-acquisition analysis because of clipping in more than 50% data or non-discernible UR. We then filtered SCR data with a first-order bidirectional band-pass Butterworth filter with a cut-off frequency of 0.0159-5 Hz and down-sampled the data to 10 Hz (5). We used a forward non-linear psychophysiological model in PsPM (5–7) to estimate CS-elicited SCR amplitudes for each trial. The model comprised a CS-evoked fixed-latency neural burst with 0.3-s dispersion and a flexible-latency burst between CS onset and 1.5 s before US time point with fixed dispersion of 0.3 s; both were convolved with a canonical SCR function (5,8). Based on methodological work (9), only the fixed-latency SCR amplitudes were retained for further statistical analysis and normalized for each participant by dividing through the average of all CS- trials for this participant.

Pupil dilation

Pupil diameter was converted from arbitrary units to millimeters, pre-processed and combined from both eyes in PsPM (10,11). We corrected apparent pupil size for gaze deviation (12) due to the full-screen CSs, which exceeded foveal vision and thus required gaze movements. Pupil data with gaze coordinates outside 9.2654° visual angle, corresponding to the entire vertical extension of the screen and the horizontal area of interest in the room images, were excluded. We then estimated pupil dilation for each trial in a standard GLM approach using a canonical fear-conditioning pupil response function in PsPM (10,13). To account for different luminance of the CS images, the model included the predicted luminance response time series as nuisance regressors (10). We excluded trials with more than 50% missing data during CS presentation, and participants with more than 50% missing trials were excluded from further analysis. This excluded two females in the minocycline group and four females and two males in the placebo group for acquisition training, four females in the minocycline group and five females and three males in the placebo group for the recall test, and four females and one male in the minocycline group and six females and three males in the placebo group for re-acquisition training. For the remaining participants, more than 90.4% of the data were included in the analysis.

## Supplementary Figures


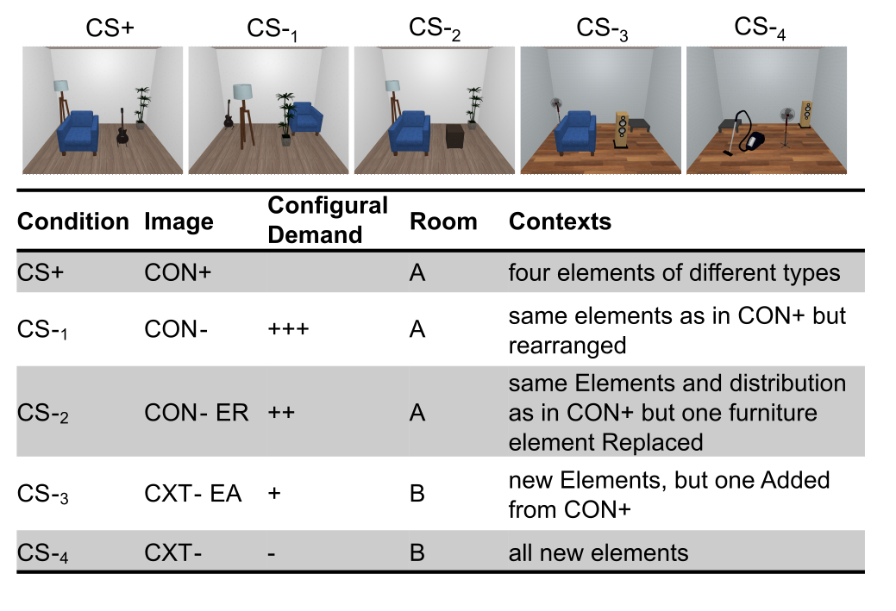


Figure S1. Static CS images and the summary table of the design and assignment of the room images (14,15). CS+ was always the CON+ image for every participant, and all the other images were CS-. All participants were presented with an overview of all CSs on the same screen before the acquisition training started for familiarization; the position of each image on the screen was randomized during this familiarization phase. CS images were presented full-screen during the experiment.


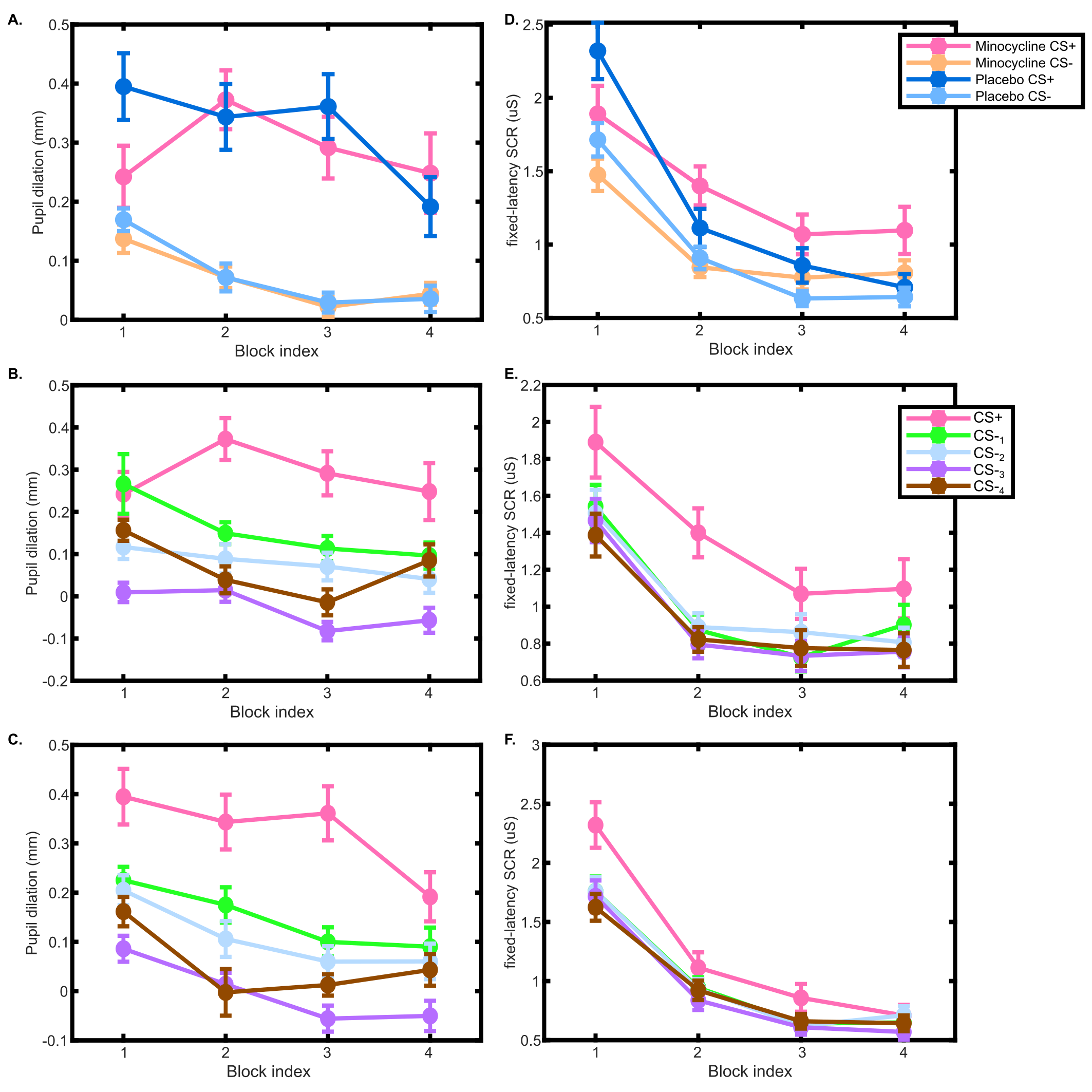


Figure S2. Block-wise pupil dilation **(A-C)** and normalized fixed-latency SCR (**D-F**) in the acquisition training. **B,E,** data from participants treated with minocycline; **C,F,** data from participants treated with the placebo. Data presented are the group means ± SEM of each block in each condition.


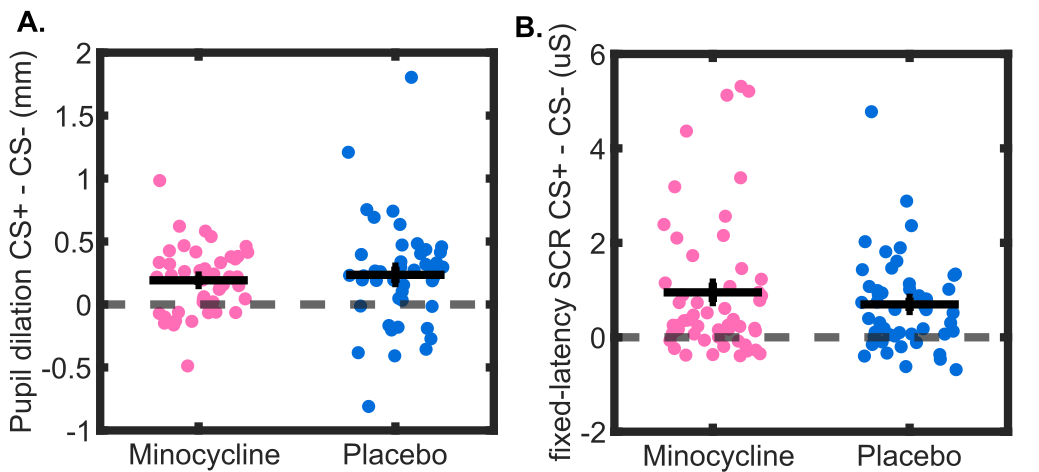


Figure S3. Configural fear memory re-acquisition. (**A**) Pupil dilation and (**B**) normalized fixed-latency SCR to CS onset in the re-acquisition training. Only data from non-reinforced trials are included. Black crosses represent the group means ± SEM of the difference between CS+ and averaged CS- over all four CS- conditions.


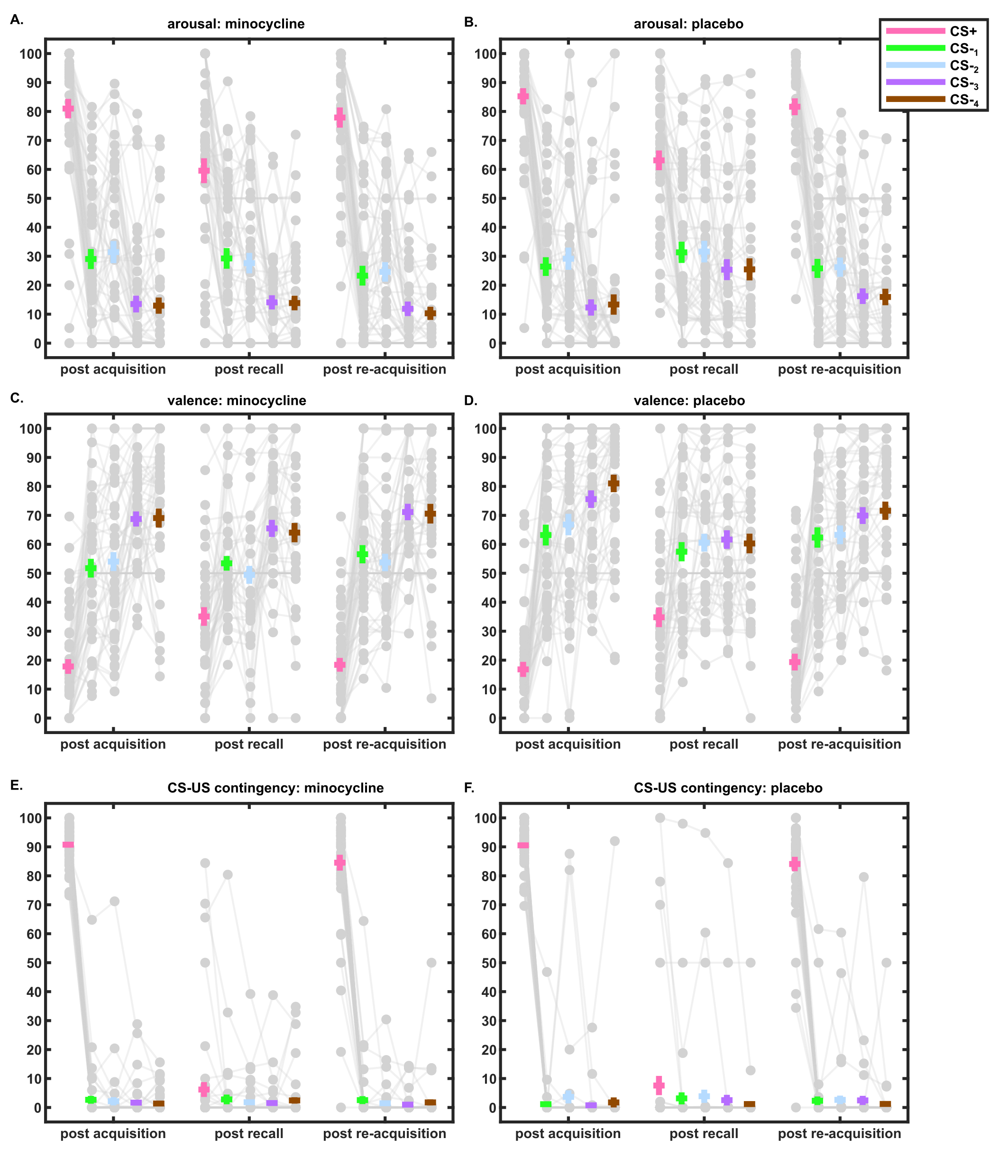


Figure S4. Subjective ratings of arousal, valence and CS-US contingency. Individual data are plotted in grey and group data in the corresponding colors with group means and SEM of each condition.

## Supplementary Tables

Table S1. Sample sizes in each analysis

|  |  | **minocycline** | | **placebo** | |
| --- | --- | --- | --- | --- | --- |
|  |  | female | male | female | male |
| memory acquisition | pupil dilation | 33 | 18 | 31 | 17 |
|  | SCR | 35 | 18 | 33 | 19 |
| memory retention | FPS | 35 | 17 | 35 | 18 |
|  | pupil dilation | 31 | 17 | 30 | 15 |
| memory re-acquisition | pupil dilation | 31 | 16 | 29 | 15 |
|  | SCR | 35 | 17 | 33 | 17 |

Table S2. The summary table of FPS LME results for memory recall test

| **Effect** | **Estimate** | **df** | **t** | ***p*** |
| --- | --- | --- | --- | --- |
| Intercept | 1.59 | 9115 | 59.815 | **< .001** |
| Drug Minocycline | -0.1514 | 9115 | -4.01 | **< .001** |
| CS-1 | -0.2579 | 9115 | -6.3 | **< .001** |
| CS-2 | -0.1249 | 9115 | -3.05 | **0.002** |
| CS-3 | -0.1274 | 9115 | -3.12 | **0.002** |
| CS-4 | -0.1771 | 9115 | -4.34 | **< .001** |
| Trial Index | -0.013 | 9115 | -25.82 | **< .001** |
| Drug Minocycline x CS-1 | 0.1906 | 9115 | 3.27 | **0.001** |
| Drug Minocycline x CS-2 | 0.0364 | 9115 | 0.63 | 0.531 |
| Drug Minocycline x CS-3 | 0.1217 | 9115 | 2.09 | **0.036** |
| Drug Minocycline x CS-4 | 0.1282 | 9115 | 2.21 | **0.027** |
| Drug Minocycline x Trial Index | 0.0032 | 9115 | 4.44 | **< .001** |
| CS-1 x Trial Index | 0.0045 | 9115 | 5.62 | **< .001** |
| CS-2 x Trial Index | 0.0021 | 9115 | 2.62 | **0.009** |
| CS-3 x Trial Index | 0.0028 | 9115 | 3.47 | **0.001** |
| CS-4 x Trial Index | 0.0034 | 9115 | 4.25 | **< .001** |
| Drug Minocycline x CS-1 x Trial Index | -0.0033 | 9115 | -2.88 | **0.004** |
| Drug Minocycline x CS-2 x Trial Index | -0.0007 | 9115 | -0.6 | 0.552 |
| Drug Minocycline x CS-3 x Trial Index | -0.0028 | 9115 | -2.47 | **0.013** |
| Drug Minocycline x CS-4 x Trial Index | -0.0024 | 9115 | -2.16 | **0.031** |

Table S3. T-test results for memory retention

| **Measure** | **Group** | **df** | **t** | ***p*** | **cohens' d** | **CI 95%** |
| --- | --- | --- | --- | --- | --- | --- |
| FPS  Subset 1 | Placebo | 51 | 5.26 | **< .001** | 0.73 | [0.30, 0.67] |
|  | Minocycline | 50 | 0.78 | .44 | 0.11 | [-0.09, 0.21] |
|  | Mino vs. Placebo | 101 | -3.62 | **< .001** | -0.71 | [-0.67, -0.19] |
| FPS  Subset 1-2 | Placebo | 52 | 4.32 | **< .001** | 0.59 | [0.16, 0.45] |
|  | Minocycline | 51 | 2.29 | .053 | 0.32 | [0.01, 0.20] |
|  | Mino vs. Placebo | 103 | -2.33 | **.022** | -0.46 | [-0.37, -0.03] |
| Pupil dilation  Subset 1 | Placebo | 41 | 6.85 | **< .001** | 1.06 | [0.24, 0.44] |
|  | Minocycline | 43 | 4.84 | **< .001** | 0.73 | [0.16, 0.40] |
|  | Mino vs. Placebo | 84 | -0.73 | .653 | -0.16 | [-0.21, 0.10] |
| Pupil dilation  Subset 1-2 | Placebo | 43 | 6.61 | **< .001** | 1.00 | [0.22, 0.42] |
|  | Minocycline | 44 | 4.80 | **< .001** | 0.72 | [0.15, 0.36] |
|  | Mino vs. Placebo | 87 | -0.99 | .653 | -0.21 | [-0.21, 0.07] |

Note: P-values of memory retention analyses were Holm-Bonferroni corrected for two comparisons (i.e., subset 1 and subset 1-2). Mino, minocycline. Paired t-tests for within-group CS+/- difference and unpaired t-test for between-group difference.

Table S4. The summary table of pupil dilation LME results for memory acquisition

| Effect | Estimate | df | t | p |
| --- | --- | --- | --- | --- |
| Intercept | 0.439 | 5408 | 8.09 | **< .001** |
| Drug Minocycline | -0.1478 | 5420 | -1.95 | 0.052 |
| CS-1 | -0.1827 | 5921 | -3.05 | **0.002** |
| CS-2 | -0.2055 | 5923 | -3.42 | **0.001** |
| CS-3 | -0.3146 | 5922 | -5.24 | **< .001** |
| CS-4 | -0.2849 | 5922 | -4.75 | **< .001** |
| Trial Index | -0.0029 | 5924 | -2.55 | **0.011** |
| Drug Minocycline x CS-1 | 0.1667 | 5922 | 1.99 | **0.047** |
| Drug Minocycline x CS-2 | 0.0549 | 5923 | 0.65 | 0.514 |
| Drug Minocycline x CS-3 | 0.0825 | 5922 | 0.98 | 0.327 |
| Drug Minocycline x CS-4 | 0.1401 | 5923 | 1.67 | 0.096 |
| Drug Minocycline x Trial Index | 0.0028 | 5926 | 1.79 | 0.074 |
| CS-1 x Trial Index | 0.0004 | 5922 | 0.35 | 0.723 |
| CS-2 x Trial Index | 0.00003 | 5925 | 0.03 | 0.979 |
| CS-3 x Trial Index | -0.00005 | 5923 | -0.04 | 0.968 |
| CS-4 x Trial Index | 0.00067 | 5923 | 0.53 | 0.596 |
| Drug Minocycline x CS-1 x Trial Index | -0.0031 | 5924 | -1.76 | 0.078 |
| Drug Minocycline x CS-2 x Trial Index | -0.0013 | 5925 | -0.73 | 0.466 |
| Drug Minocycline x CS-3 x Trial Index | -0.0018 | 5923 | -1.01 | 0.315 |
| Drug Minocycline x CS-4 x Trial Index | -0.0024 | 5924 | -1.36 | 0.175 |

Table S5. The summary table of SCR LME results for memory acquisition

| Effect | Estimate | df | t | p |
| --- | --- | --- | --- | --- |
| Intercept | 2.214 | 7021 | 18.82 | **< .001** |
| Drug Minocycline | -0.344 | 7021 | -2.09 | **0.037** |
| CS-1 | -0.501 | 7021 | -3.78 | **< .001** |
| CS-2 | -0.509 | 7021 | -3.84 | **< .001** |
| CS-3 | -0.54 | 7021 | -4.07 | **< .001** |
| CS-4 | -0.622 | 7021 | -4.69 | **< .001** |
| Trial Index | -0.024 | 7021 | -9.55 | **< .001** |
| Drug Minocycline x CS-1 | 0.091 | 7021 | 0.49 | 0.625 |
| Drug Minocycline x CS-2 | 0.092 | 7021 | 0.49 | 0.622 |
| Drug Minocycline x CS-3 | 0.048 | 7021 | 0.26 | 0.795 |
| Drug Minocycline x CS-4 | 0.111 | 7021 | 0.6 | 0.55 |
| Drug Minocycline x Trial Index | 0.011 | 7021 | 3.18 | **0.001** |
| CS-1 x Trial Index | 0.007 | 7021 | 2.69 | **0.007** |
| CS-2 x Trial Index | 0.008 | 7021 | 2.77 | **0.006** |
| CS-3 x Trial Index | 0.007 | 7021 | 2.45 | **0.014** |
| CS-4 x Trial Index | 0.009 | 7021 | 3.32 | **0.001** |
| Drug Minocycline x CS-1 x Trial Index | -0.005 | 7021 | -1.35 | 0.176 |
| Drug Minocycline x CS-2 x Trial Index | -0.005 | 7021 | -1.35 | 0.176 |
| Drug Minocycline x CS-3 x Trial Index | -0.005 | 7021 | -1.17 | 0.242 |
| Drug Minocycline x CS-4 x Trial Index | -0.006 | 7021 | -1.65 | 0.099 |

Table S6. T-test results for memory re-acquisition

| **Measure** | **Group** | **df** | **t** | ***p*** | **cohens' d** | **CI 95%** |
| --- | --- | --- | --- | --- | --- | --- |
| Pupil dilation | Placebo | 43 | 3.60 | **< .001** | 0.54 | [0.10, 0.37] |
|  | Minocycline | 46 | 5.13 | **< .001** | 0.75 | [0.12, 0.27] |
|  | Mino vs. Placebo | 89 | -0.58 | 0.562 | -0.12 | [-0.19, 0.10] |
| SCR | Placebo | 49 | 5.00 | **< .001** | 0.71 | [0.42, 0.98] |
|  | Minocycline | 51 | 4.62 | **< .001** | 0.64 | [0.54, 1.36] |
|  | Mino vs. Placebo | 100 | 1.02 | 0.311 | 0.20 | [-0.24, 0.75] |

Note: Mino, minocycline. Paired t-tests for within-group CS+/- difference and unpaired t-test for between-group difference.

Table S7. LME results for memory re-acquisition

|  | **Memory re-acquisition** | | | | | | | | |
| --- | --- | --- | --- | --- | --- | --- | --- | --- | --- |
|  | **Pupil dilation** | | | |  | **Fixed-latency SCR** | | | |
| Effect | F |  | df | *p* |  | F |  | df | *p* |
| Drug (placebo/minocycline) | 2.41 |  | 1, 2817.1 | .121 |  | 0.01 |  | 1, 3395 | .908 |
| Trial index | 26.83 |  | 1, 2748.0 | **< .001** |  | 160.32 |  | 1, 3395 | **< .001** |
| Condition (CS+/CS-_1,2,3,4_) | 8.82 |  | 4, 2744.5 | **< .001** |  | 4.23 |  | 4, 3395 | **.002** |
| Drug x Trial index | 1.59 |  | 1, 2748.0 | .207 |  | 0.01 |  | 1, 3395 | .925 |
| Drug x Condition | 0.90 |  | 4, 2744.5 | .464 |  | 0.35 |  | 4, 3395 | .846 |
| Trial index x Condition | 6.13 |  | 4, 2744.6 | **< .001** |  | 2.21 |  | 4, 3395 | .066 |
| Drug x Trial index x Condition | 0.78 |  | 4, 2744.6 | .535 |  | 0.43 |  | 4, 3395 | .786 |

Table S8. rm-ANOVA results for subjective ratings

|  | **Arousal** | | | | |
| --- | --- | --- | --- | --- | --- |
| Effect | F |  | df | p | η² |
| Drug (placebo/minocycline) | 1.57 |  | 1, 103 | .213 | 0.015 |
| Rating time | 0.93 |  | 1.75, 180.32 | .380 | 0.009 |
| Condition | 259.15 |  | 2.49, 256.17 | **< .001** | 0.716 |
| Drug x Rating time | 2.52 |  | 1.75, 180.32 | .090 | 0.024 |
| Drug x Condition | 0.56 |  | 2.49, 256.17 | .607 | 0.005 |
| Rating time x Condition | 22.14 |  | 5.13, 528.01 | **< .001** | 0.177 |
| Drug x Rating time x Condition | 0.97 |  | 5.13, 528.01 | .438 | 0.009 |
|  | **Valence** | | | | |
| Effect | F |  | df | p | η² |
| Drug (placebo/minocycline) | 3.57 |  | 1, 103 | .062 | 0.033 |
| Rating time | 1.36 |  | 1.81, 186.68 | .267 | 0.013 |
| Condition | 152.36 |  | 2.34, 241.35 | **< .001** | 0.597 |
| Drug x Rating time | 3.28 |  | 1.81, 186.68 | **.044** | 0.031 |
| Drug x Condition | 2.44 |  | 2.34, 241.35 | .080 | 0.023 |
| Rating time x Condition | 21.41 |  | 5.97, 614.79 | **< .001** | 0.172 |
| Drug x Rating time x Condition | 1.59 |  | 5.97, 614.79 | .147 | 0.015 |
|  | **CS-US contingency** | | | | |
| Effect | F |  | df | p | η² |
| Drug (placebo/minocycline) | 0.06 |  | 1, 103 | .813 | 0.001 |
| Rating time | 203.59 |  | 1.58, 162.86 | **< .001** | 0.664 |
| Condition | 2039.23 |  | 2.75, 283.71 | **< .001** | 0.952 |
| Drug x Rating time | 0.12 |  | 1.58, 162.86 | .843 | 0.001 |
| Drug x Condition | 0.53 |  | 2.75, 283.71 | .644 | 0.005 |
| Rating time x Condition | 561.99 |  | 3.63, 374.19 | **< .001** | 0.845 |
| Drug x Rating time x Condition | 0.28 |  | 3.63, 374.19 | .878 | 0.003 |

Table S9. LME results in female participants

|  | **Female** | | | | | | | | |
| --- | --- | --- | --- | --- | --- | --- | --- | --- | --- |
|  | **Memory acquisition** | | | | | | | | |
|  | **Pupil dilation** | | | |  | **Fixed-latency SCR** | | | |
| Effect | F |  | df | *p* |  | F |  | df | *p* |
| Drug (placebo/minocycline) | 4.50 |  | 1, 223.6 | **.035** |  | 17.71 |  | 1, 1074.9 | **< .001** |
| Trial index | 61.04 |  | 1, 3783.5 | **< .001** |  | 652.54 |  | 1, 4465.0 | **< .001** |
| Condition (CS+/CS-_1,2,3,4_) | 12.23 |  | 4, 3768.7 | **< .001** |  | 7.18 |  | 4, 4461.2 | **< .001** |
| Drug x Trial index | 6.88 |  | 1, 3783.5 | **.009** |  | 31.02 |  | 1, 4465.0 | **< .001** |
| Drug x Condition | 0.72 |  | 4, 3768.7 | .58 |  | 0.23 |  | 4, 4461.2 | .92 |
| Trial index x Condition | 1.07 |  | 4, 3769.8 | .37 |  | 2.41 |  | 4, 4462.5 | **.047** |
| Drug x Trial index x Condition | 0.46 |  | 4, 3769.8 | .76 |  | 0.09 |  | 4, 4462.5 | .99 |
|  | **Memory retention** | | | | | | | | |
|  | **FPS** | | | |  | **Pupil dilation** | | | |
| Effect | F |  | df | *p* |  | F |  | df | *p* |
| Drug (placebo/minocycline) | 28.55 |  | 1, 864.2 | **< .001** |  | 0.60 |  | 1, 193.7 | .44 |
| Trial index | 1951.64 |  | 1, 6001.6 | **< .001** |  | 116.72 |  | 1, 4789.6 | **< .001** |
| Condition (CS+/CS-_1,2,3,4_) | 6.51 |  | 4, 5989.5 | **< .001** |  | 6.75 |  | 4, 4764.9 | **< .001** |
| Drug x Trial index | 40.40 |  | 1, 6001.6 | **< .001** |  | 0.71 |  | 1, 4789.6 | .40 |
| Drug x Condition | 3.81 |  | 4, 5989.5 | **.004** |  | 2.66 |  | 4, 4764.9 | **.031** |
| Trial index x Condition | 4.97 |  | 4, 5990.4 | **< .001** |  | 1.84 |  | 4, 4765.8 | .12 |
| Drug x Trial index x Condition | 3.16 |  | 4, 5990.4 | **.013** |  | 1.29 |  | 4, 4765.8 | .27 |
|  | **Memory re-acquisition** | | | | | | | | |
|  | **Pupil dilation** | | | |  | **Fixed-latency SCR** | | | |
| Effect | F |  | df | *p* |  | F |  | df | *p* |
| Drug (placebo/minocycline) | 2.58 |  | 1, 1829.2 | .11 |  | 6.43 |  | 1, 2251 | **.011** |
| Trial index | 25.53 |  | 1, 1786.8 | **< .001** |  | 63.15 |  | 1, 2251 | **< .001** |
| Condition (CS+/CS-_1,2,3,4_) | 5.56 |  | 4, 1784.0 | **< .001** |  | 1.76 |  | 4, 2251 | .13 |
| Drug x Trial index | 2.04 |  | 1, 1786.8 | .15 |  | 7.15 |  | 1, 2251 | **.008** |
| Drug x Condition | 0.87 |  | 4, 1784.0 | .48 |  | 0.94 |  | 4, 2251 | .44 |
| Trial index x Condition | 4.35 |  | 4, 1784.1 | **.002** |  | 0.80 |  | 4, 2251 | .53 |
| Drug x Trial index x Condition | 0.71 |  | 4, 1784.1 | .58 |  | 1.01 |  | 4, 2251 | .40 |

Table S10. T-test results in female participants

| **Measure** | **Group** | **df** | **t** | ***p*** | **cohens' d** | **CI 95%** |
| --- | --- | --- | --- | --- | --- | --- |
| Memory acquisition: Pupil dilation | Placebo | 30 | 6.24 | **< .001** | 1.12 | [0.18, 0.35] |
|  | Minocycline | 32 | 3.64 | **< .001** | 0.63 | [0.08, 0.28] |
|  | Mino vs. Placebo | 62 | -1.34 | 0.186 | -0.33 | [-0.22, 0.04] |
| Memory acquisition: SCR | Placebo | 32 | 2.68 | **0.011** | 0.47 | [0.06, 0.41] |
|  | Minocycline | 34 | 4.79 | **< .001** | 0.81 | [0.23, 0.57] |
|  | Mino vs. Placebo | 66 | 1.37 | 0.174 | 0.33 | [-0.08, 0.41] |
| Memory retention: FPS subset 1 | Placebo | 33 | 5.31 | **< .001** | 0.91 | [0.37, 0.83] |
|  | Minocycline | 33 | -0.06 | 0.949 | -0.01 | [-0.17, 0.17] |
|  | Mino vs. Placebo | 66 | -4.32 | **< .001** | -1.05 | [-0.88, -0.32] |
| Memory retention: FPS subset 1-2 | Placebo | 34 | 3.93 | **< .001** | 0.66 | [0.17, 0.55] |
|  | Minocycline | 34 | 1.31 | 0.397 | 0.22 | [-0.04, 0.19] |
|  | Mino vs. Placebo | 68 | -2.69 | **0.009** | -0.64 | [-0.50, -0.07] |
| Memory retention: Pupil dilation subset 1 | Placebo | 26 | 5.81 | **< .001** | 1.12 | [0.24, 0.50] |
|  | Minocycline | 27 | 3.11 | **0.007** | 0.59 | [0.08, 0.38] |
|  | Mino vs. Placebo | 53 | -1.40 | 0.311 | -0.38 | [-0.34, 0.06] |
| Memory retention: Pupil dilation subset 1-2 | Placebo | 28 | 5.17 | **< .001** | 0.96 | [0.20, 0.47] |
|  | Minocycline | 28 | 3.21 | **0.007** | 0.60 | [0.07, 0.33] |
|  | Mino vs. Placebo | 56 | -1.44 | 0.311 | -0.38 | [-0.31, 0.05] |
| Memory re-acquisition: Pupil dilation | Placebo | 28 | 3.40 | **0.002** | 0.63 | [0.12, 0.48] |
|  | Minocycline | 30 | 2.72 | **0.011** | 0.49 | [0.03, 0.19] |
|  | Mino vs. Placebo | 58 | -1.97 | 0.054 | -0.51 | [-0.37, 0.003] |
| Memory re-acquisition: SCR | Placebo | 32 | 3.65 | **< .001** | 0.64 | [0.31, 1.09] |
|  | Minocycline | 34 | 3.48 | **0.001** | 0.59 | [0.41, 1.55] |
|  | Mino vs. Placebo | 66 | 0.81 | 0.418 | 0.20 | [-0.41, 0.97] |

Note: P-values of memory retention analyses were Holm-Bonferroni corrected for two comparisons (i.e., subset 1 and subset 1-2). Mino, minocycline. Paired t-tests for within-group CS+/- difference and unpaired t-test for between-group difference.

Table S11. LME results in male participants

|  | **Male** | | | | | | | | |
| --- | --- | --- | --- | --- | --- | --- | --- | --- | --- |
|  | **Memory acquisition** | | | | | | | | |
|  | **Pupil dilation** | | | |  | **Fixed-latency SCR** | | | |
| Effect | F |  | df | *p* |  | F |  | df | *p* |
| Drug (placebo/minocycline) | 0.69 |  | 1, 246.7 | .41 |  | 16.76 |  | 1, 2467 | **< .001** |
| Trial index | 29.08 |  | 1, 2140.3 | **< .001** |  | 197.85 |  | 1, 2467 | **< .001** |
| Condition (CS+/CS-_1,2,3,4_) | 4.93 |  | 4, 2136.8 | **< .001** |  | 2.60 |  | 4, 2467 | **.034** |
| Drug x Trial index | 0.44 |  | 1, 2140.3 | .51 |  | 19.59 |  | 1, 2467 | **< .001** |
| Drug x Condition | 2.36 |  | 4, 2136.8 | .051 |  | 1.35 |  | 4, 2467 | .25 |
| Trial index x Condition | 0.57 |  | 4, 2137.4 | .68 |  | 0.35 |  | 4, 2467 | .84 |
| Drug x Trial index x Condition | 3.76 |  | 4, 2137.4 | **.005** |  | 1.78 |  | 4, 2467 | .13 |
|  | **Memory retention** | | | | | | | | |
|  | **FPS** | | | |  | **Pupil dilation** | | | |
| Effect | F |  | df | *p* |  | F |  | df | *p* |
| Drug (placebo/minocycline) | 7.63 |  | 1, 3036 | **.006** |  | 0.15 |  | 1, 66.7 | .70 |
| Trial index | 765.9 |  | 1, 3036 | **< .001** |  | 14.56 |  | 1, 2541.8 | **< .001** |
| Condition (CS+/CS-_1,2,3,4_) | 3.21 |  | 4, 3036 | **.012** |  | 4.47 |  | 4, 2538.8 | **.001** |
| Drug x Trial index | 9.16 |  | 1, 3036 | **.002** |  | 0.21 |  | 1, 2541.8 | .65 |
| Drug x Condition | 0.50 |  | 4, 3036 | .74 |  | 0.20 |  | 4, 2538.8 | .94 |
| Trial index x Condition | 3.38 |  | 4, 3036 | **.009** |  | 1.93 |  | 4, 2539.0 | .10 |
| Drug x Trial index x Condition | 0.56 |  | 4, 3036 | .69 |  | 0.56 |  | 4, 2539.0 | .69 |
|  | **Memory re-acquisition** | | | | | | | | |
|  | **Pupil dilation** | | | |  | **Fixed-latency SCR** | | | |
| Effect | F |  | df | *p* |  | F |  | df | *p* |
| Drug (placebo/minocycline) | 0.17 |  | 1. 966.8 | .68 |  | 17.42 |  | 1, 1124 | **< .001** |
| Trial index | 3.13 |  | 1, 942.9 | .077 |  | 125.21 |  | 1, 1124 | **< .001** |
| Condition (CS+/CS-_1,2,3,4_) | 4.00 |  | 4, 942.4 | **.003** |  | 2.91 |  | 4, 1124 | **.021** |
| Drug x Trial index | 0.03 |  | 1, 942.9 | .87 |  | 15.97 |  | 1, 1124 | **< .001** |
| Drug x Condition | 0.35 |  | 4, 942.4 | .84 |  | 0.90 |  | 4, 1124 | .46 |
| Trial index x Condition | 2.66 |  | 4, 942.4 | **.031** |  | 1.75 |  | 4, 1124 | .14 |
| Drug x Trial index x Condition | 0.23 |  | 4, 942.4 | .92 |  | 0.84 |  | 4, 1124 | .50 |

Table S12. T-test results in male participants

| **Measure** | **Group** | **df** | **t** | ***p*** | **cohens' d** | **CI 95%** |
| --- | --- | --- | --- | --- | --- | --- |
| Memory acquisition: Pupil dilation | Placebo | 16 | 3.85 | **0.001** | 0.93 | [0.09, 0.31] |
|  | Minocycline | 17 | 5.07 | **< .001** | 1.20 | [0.16, 0.38] |
|  | Mino vs. Placebo | 33 | 0.85 | 0.404 | 0.29 | [-0.09, 0.21] |
| Memory acquisition: SCR | Placebo | 18 | 4.05 | **< .001** | 0.93 | [0.18, 0.56] |
|  | Minocycline | 17 | 3.93 | **0.001** | 0.93 | [0.19, 0.64] |
|  | Mino vs. Placebo | 35 | 0.37 | 0.717 | 0.12 | [-0.23, 0.33] |
| Memory retention: FPS subset 1 | Placebo | 17 | 1.81 | 0.156 | 0.43 | [-0.05, 0.61] |
|  | Minocycline | 16 | 1.24 | 0.234 | 0.30 | [-0.13, 0.50] |
|  | Mino vs. Placebo | 33 | -0.45 | 1 | -0.15 | [-0.53, 0.34] |
| Memory retention: FPS subset 1-2 | Placebo | 17 | 1.87 | 0.156 | 0.44 | [-0.02, 0.40] |
|  | Minocycline | 16 | 2.07 | 0.109 | 0.50 | [-0.004, 0.36] |
|  | Mino vs. Placebo | 33 | -0.11 | 1 | -0.04 | [-0.29, 0.26] |
| Memory retention: Pupil dilation subset 1 | Placebo | 14 | 3.61 | **0.003** | 0.93 | [0.11, 0.45] |
|  | Minocycline | 15 | 4.02 | **0.002** | 1.00 | [0.17, 0.57] |
|  | Mino vs. Placebo | 29 | 0.72 | 0.953 | 0.26 | [-0.16, 0.34] |
| Memory retention: Pupil dilation subset 1-2 | Placebo | 14 | 4.19 | **0.002** | 1.08 | [0.14, 0.44] |
|  | Minocycline | 15 | 3.74 | **0.002** | 0.93 | [0.14, 0.52] |
|  | Mino vs. Placebo | 29 | 0.36 | 0.953 | 0.13 | [-0.19, 0.28] |
| Memory re-acquisition: Pupil dilation | Placebo | 14 | 1.36 | 0.196 | 0.35 | [-0.07, 0.30] |
|  | Minocycline | 15 | 5.76 | **< .001** | 1.44 | [0.22, 0.48] |
|  | Mino vs. Placebo | 29 | 2.23 | **0.034** | 0.80 | [0.02, 0.45] |
| Memory re-acquisition: SCR | Placebo | 16 | 3.86 | **0.001** | 0.94 | [0.31, 1.07] |
|  | Minocycline | 16 | 3.46 | **0.003** | 0.84 | [0.34, 1.43] |
|  | Mino vs. Placebo | 32 | 0.64 | 0.525 | 0.22 | [-0.44, 0.84] |

Note: P-values of memory retention analyses were Holm-Bonferroni corrected for two comparisons (i.e., subset 1 and subset 1-2). Mino, minocycline. Paired t-tests for within-group CS+/- difference and unpaired t-test for between-group difference.

## References in the supplementary information:

1. Mathôt S, Schreij D, Theeuwes J. OpenSesame: an open-source, graphical experiment builder for the social sciences. Behav Res Methods. 2012 Jun;44(2):314–24.

2. Hygge S, Hugdahl K. Skin Conductance Recordings and the NaCl Concentration of the Electrolyte. Psychophysiology. 1985 May;22(3):365–7.

3. Blumenthal TD, Cuthbert BN, Filion DL, Hackley S, Lipp OV, Van Boxtel A. Committee report: Guidelines for human startle eyeblink electromyographic studies. Psychophysiology. 2005 Jan;42(1):1–15.

4. Khemka S, Tzovara A, Gerster S, Quednow BB, Bach DR. Modeling startle eyeblink electromyogram to assess fear learning: Modeling startle-blink EMG to assess fear learning. Psychophysiol. 2017 Feb;54(2):204–14.

5. Staib M, Castegnetti G, Bach DR. Optimising a model-based approach to inferring fear learning from skin conductance responses. Journal of Neuroscience Methods. 2015 Nov;255:131–8.

6. Bach DR, Daunizeau J, Friston KJ, Dolan RJ. Dynamic causal modelling of anticipatory skin conductance responses. Biological Psychology. 2010 Sep;85(1):163–70.

7. Gerster S, Namer B, Elam M, Bach DR. Testing a linear time invariant model for skin conductance responses by intraneural recording and stimulation. Psychophysiology [Internet]. 2018;55(2). Available from: https://www.ncbi.nlm.nih.gov/pubmed/28862764

8. Bach DR, Flandin G, Friston KJ, Dolan RJ. Modelling event-related skin conductance responses. Int J Psychophysiol. 2010 Mar;75(3):349–56.

9. Xia Y, Wehrli J, Gerster S, Kroes M, Houtekamer M, Bach DR. Measuring human context fear conditioning and retention after consolidation. Learn Mem. 2023 Jul;30(7):139–50.

10. Korn CW, Bach DR. A solid frame for the window on cognition: Modeling event-related pupil responses. Journal of Vision. 2016 Feb;16(3):28.

11. Kret ME, Sjak-Shie EE. Preprocessing pupil size data: Guidelines and code. Behavior research methods. 2019;1–7.

12. Hayes TR, Petrov AA. Mapping and correcting the influence of gaze position on pupil size measurements. Behav Res. 2016 Jun;48(2):510–27.

13. Korn CW, Staib M, Tzovara A, Castegnetti G, Bach DR. A pupil size response model to assess fear learning: Pupil responses and fear conditioning. Psychophysiol. 2017 Mar;54(3):330–43.

14. Stout DM, Glenn DE, Acheson DT, Spadoni AD, Risbrough VB, Simmons AN. Neural measures associated with configural threat acquisition. Neurobiology of Learning and Memory. 2018 Apr;150:99–106.

15. Stout DM, Glenn DE, Acheson DT, Simmons AN, Risbrough VB. Characterizing the neural circuitry associated with configural threat learning. Brain Res. 2019 Sep 15;1719:225–34.
